# The impact of multicenter microbiology laboratory workflow optimization on antimicrobial susceptibility testing reporting time and outcomes of patients with bloodstream infection

**DOI:** 10.3389/fcimb.2026.1875923

**Published:** 2026-09-16

**Authors:** Jing Shao, Shuhong Sun, Lijuan Liu, Jing Li, Renzhe Li, Haixin Dong, Yujie Wang, Guixia Li, Chunzhong Dong, Maoli Yi, Yan Jin

**Affiliations:** 1 Department of Clinical Laboratory, Shandong Provincial Hospital Affiliated to Shandong First Medical University, Jinan, Shandong, China; 2 Department of Laboratory Medicine, Linyi People’s Hospital, Linyi, Shandong, China; 3 Department of Clinical Laboratory, The Affiliated People’s Hospital of Shandong First Medical University, Jinan, Shandong, China; 4 Department of Clinical Laboratory, The Afffliated Hospital of Qingdao University, Qingdao, Shandong, China; 5 Department of Clinical Laboratory, Jining No.1 People’s Hospital, Jining, Shandong, China; 6 Department of Clinical Laboratory, Affiliated Hospital of Jining Medical University, Jining, Shandong, China; 7 Department of Clinical Laboratory, Qingdao Municipal Hospital, Qingdao, Shandong, China; 8 The Laboratory Department of Heze Municipal Hospital, Heze, Shandong, China; 9 Department of Clinical Laboratory, Binzhou People’s Hospital, Binzhou, Shandong, China; 10 Department of Clinical Laboratory, Yantai Yuhuangding Hospital, Yantai, Shandong, China

**Keywords:** antimicrobial susceptibility testing, blood culture, bloodstream infection, multicenter study, turnaround time, workflow optimization

## Abstract

Bloodstream infections (BSIs) are life-threatening disorders linked to substantial global mortality, and timely pathogen identification (ID) and antimicrobial susceptibility testing (AST) are indispensable for targeted clinical management. Blood culture (BC) remains the gold-standard diagnostic approach, but conventional workflows often lead to prolonged turnaround times (TAT) and delayed targeted therapy. This multicenter retrospective study aimed to evaluate the impact of the BC workflow optimization strategy in 10 tertiary hospitals in Shandong Province, China. The intervention included 24/7 uninterrupted specimen processing, rapid MALDI-TOF MS ID after short incubation periods of 6–8 h with results reporting, and second-day MALDI-TOF MS ID post-BC positivity with results reporting. A total of 1, 826 adult BSI patients were enrolled, with 942 in the pre-optimization group and 884 in the post-optimization group. The primary outcomes were BC TAT metrics, while secondary outcomes included pathogen distribution, antibiotic use, clinical outcomes, and healthcare costs. Compared with the pre-optimization group, the time from blood collection to the reporting of Gram-stain, MALDI-TOF MS ID and final AST were reduced by 13.4 h, 11.0 h and 18.5 h, respectively, in the optimized group. The median AST TAT for major pathogens decreased from 70.26 h to 51.14 h. Patients in the post-optimization group exhibited lower all-cause in-hospital mortality (*P* = 0.002), lower total hospitalization costs (*P* = 0.003), and lower antimicrobial expenditure was observed after IPTW adjustment (*P* < 0.001). No significant differences were observed in hospital length of stay, laboratory testing costs or total antibiotic duration. We further performed IPTW to eliminate baseline confounding bias and reliably evaluate the independent clinical and economic benefits of this optimization bundle. This large real-world multicenter study demonstrates that BC workflow optimization substantially improves diagnostic efficiency and reduces healthcare burden, offering a replicable, cost-saving blueprint for clinical microbiology laboratories in tiered hospital systems.

## 1 Introduction

Bloodstream infections (BSIs) are rapidly progressive critical infectious disorders that can quickly progress from bacteremia to sepsis, septic shock and multiple organ failure, posing severe threats to patient survival worldwide. Epidemiological data indicate that sepsis affects approximately 40 million people globally each year, with persistently high associated morbidity and mortality (Kim et al., 2024; Qin et al., 2025). The 6-hour golden resuscitation window for sepsis patients is well-established; every one-hour delay in delivering effective targeted antimicrobial therapy reduces septic shock patient survival by 7.6% (Kumar et al., 2006; Rehn et al., 2022). Timely etiological diagnosis is therefore a core priority for optimizing BSI clinical management.

Blood culture (BC) remains the gold standard for etiological diagnosis of and the primary method for pathogen identification (ID) and antimicrobial susceptibility testing (AST) (Lamy et al., 2016). However, conventional BC workflowshave prominent drawbacks that delay diagnostic reporting: traditional daytime-only specimen reception leads to overnight sample accumulation, and routine ID relies on lengthy pure subculture (≥72 h), followed by slow AST setup. These bottlenecks substantially extend diagnostic turnaround time (TAT), resulting in prolonged empirical broad-spectrum antibiotic use, elevated antimicrobial resistance risks, and inferior patient outcomes (Kariv et al., 2013; Kadri et al., 2021; Fabre et al., 2022; Fitts et al., 2024).

Matrix-assisted laser desorption/ionization time-of-flight mass spectrometry (MALDI-TOF MS) has revolutionized microbial ID and drastically shortened diagnostic timelines relative to conventional biochemical assays (Zengin Canalp and Bayraktar, 2021; Padhy et al., 2024). A recent single-center validation confirmed that after 6–8 h short-incubation, MALDI-TOF MS achieves 99.7% species-level concordance with standard subculture ID, laying the technical foundation for rapid blood culture diagnosis (Ai et al., 2024; Dai et al., 2024; Fitts et al., 2024). Supplementary uninterrupted 24/7 specimen loading further eliminates overnight testing backlogs, a major source of pre-analytical delay in traditional laboratories. While individual workflow improvements are encouraging, key evidence gaps remain. Combined strategies 24/7 specimen processing, 6–8 h rapid MALDI-TOF ID and second-day MALDI-TOF ID have not been rigorously evaluated in multicenter real-world settings. Most studies are single-center, small-cohort or single-step designs, lacking parallel assessment of laboratory efficiency, clinical outcomes and economic impact—all critical for laboratory and stewardship planning (Holmbom et al., 2022; Squitieri et al., 2025; Park et al., 2026). No multicenter study has yet quantified the incremental TAT benefit of different intervention combinations, limiting generalizability across tiered hospital systems.

To fill the above evidence gaps, we conducted a retrospective multicenter cohort study covering 10 tertiary hospitals across 7 cities in Shandong Province, China. The primary objective was to quantify the reduction in core BC TAT metrics after implementation of the three-component optimization strategy. Secondary objectives included: (1) evaluating inter-hospital differences in AST reporting efficiency stratified by adopted optimization combinations; (2) comparing clinical outcomes and antibiotic adjustment patterns pre- versus post-optimization; (3) assessing the strategy’s impact on total hospitalization, laboratory and antimicrobial costs to provide multi-angle real-world multicenter evidence for widespread laboratory implementation of standardized rapid BC workflows.

## 2 Materials and methods

### 2.1 Study design and setting

This retrospective multicenter cohort was conducted across 10 tertiary general hospitals in 7 cities (Jinan, Linyi, Yantai, Jining, Qingdao, Heze, Binzhou) of Shandong Province, China. 7 hospitals had a bed capacity exceeding 4, 000, while the remaining 3 were mid-sized tertiary facilities with over 1, 000 beds. Uniform blood culture instruments, MALDI-TOF ID platforms and AST systems were adopted by all participating centers to minimize inter-laboratory operational variability.

The study period was divided into two phases: a pre-optimization phase (January to December in 2022) and a post-optimization phase (January to December in 2023). In January 2023, all participating hospitals selected and implemented BC workflow optimization strategies based on their own working models. During the entire study period, no major institutional adjustments were made to BSI diagnosis, treatment, or hospital infection control protocols at any participating center. Specifically, antimicrobial stewardship pre-authorization rules and audit-feedback workflows remained unchanged with no additional stewardship staff recruited; sepsis 3-hour/6-hour resuscitation bundles and empirical antibiotic guidelines were consistent across both phases; laboratory staffing only underwent shift rearrangement for night coverage without net increase of full-time employees; no laboratory information system (LIS) and electronic medical record (EMR) systems version upgrades or interface modifications were implemented; the hospital antibiotic formulary remained stable without major additions, deletions or restriction adjustments; and catheter-associated BSI prevention bundles were maintained identically. The BC workflow optimization constituted the only systematic intervention launched in 2023. We retrospectively extracted the collection time, Gram-stain report time, pathogen ID report time, and AST report time, and anonymized the relevant data for further analysis. This study was approved by the research ethics committee of Shandong Provincial Hospital Affiliated to Shandong First Medical University (No. 2021-385).

### 2.2 Study population and exclusion criteria

Eligible subjects were adult inpatients (≥ 18 years) with confirmed BSI admitted to the 10 participating hospitals during both study phases.

Inclusion criteria: Valid blood cultures meeting standardized BSI diagnostic criteria; complete electronic medical records covering clinical course, antimicrobial use, and all laboratory results.

Exclusion criteria: Repeat positive BC episodes within a single hospitalization (only the index episode retained); single-set positive skin commensals without infectious manifestations (contamination); incomplete core laboratory or economic outcome data; death within 24 h of admission before laboratory-guided intervention.

Standardized blood collection operations strictly followed CLSI M47-A2 guidelines (CLSI, 2022a). Two independent venipuncture sites were selected for each patient; one aerobic plus one anaerobic bottle constituted a single culture set, with 8–10 mL whole blood collected per set. Pre-sampling skin disinfection with 2% chlorhexidine gluconate alcohol solution was mandatory to reduce specimen contamination risk.

### 2.3 Bloodstream infections diagnostic criteria

BSI diagnosis followed the combined 2016 CLSI guidelines and CDC NHSN laboratory-confirmed bloodstream infection (LCBI) criteria, with dual laboratory-clinical adjudication. Isolation of a recognized pathogen from any single blood culture set confirmed true BSI, whereas common skin commensals required ≥2 independent positive culture sets accompanied by clinical infectious manifestations to establish diagnosis (Michels et al., 2021; Palavecino et al., 2024; Qin et al., 2025). Detailed pathogen classification and three-grade contamination adjudication rules are provided in **Supplementary Methods 1.1**.

### 2.4 BC workflow optimization strategy

All participating hospitals used the BACT/Alert 3D microbial detection system (bioMérieux, Marcy-l’Étoile, France) for BC incubation and positivity detection, the VITEK MS MALDI-TOF MS system (bioMérieux) for pathogen ID, and the VITEK 2 Compact system (bioMérieux) for AST, both before and after optimization. Sample collection, transportation, and preservation were strictly performed in compliance with CLSI M47-A2 guidelines throughout the study period (CLSI, 2022a).

The optimization strategy included three core components

- 24/7 uninterrupted specimen reception and loading (24/7 Reception): Replaced the traditional daytime-only (8:00–17:00) specimen processing model;night-shift staff were assigned to receive, register and immediately incubate all BC specimens delivered overnight, eliminating nighttime specimen backlogs.
- Rapid MALDI-TOF MS ID with result reporting: Once bottles flagged positive, a small volume of culture fluid was streaked onto sheep blood agar for short incubation (37 °C, 5% CO2 for 6–8 h). Visible early microbial growth was directly processed for MALDI-TOF ID. Results were synchronously pushed to the LIS/EMR systems for clinical reference. AST panels matching the identified species were set up within 30 min after ID, following CLSI M100 susceptibility testing standards (CLSI, 2022b). Internal validation confirmed 99.6% species-level concordance between rapid and conventional ID (**Supplementary Table 9**).
- Second-day MALDI-TOF MS ID with result reporting: Second-day routine MALDI-TOF MS ID and real-time reporting. For positive BC without sufficient early growth for rapid ID, pure colonies were harvested the next day for MALDI-TOF testing. ID results were sent to clinical staff via LIS/EMR instantly, and corresponding AST was initiated within 30 min post-ID.

Each hospital can freely choose the combination of the above optimization schemes according to its own work arrangements. The geographic distribution and optimization strategy of participating hospitals were shown in **Figure 1**.

**Figure 1:**
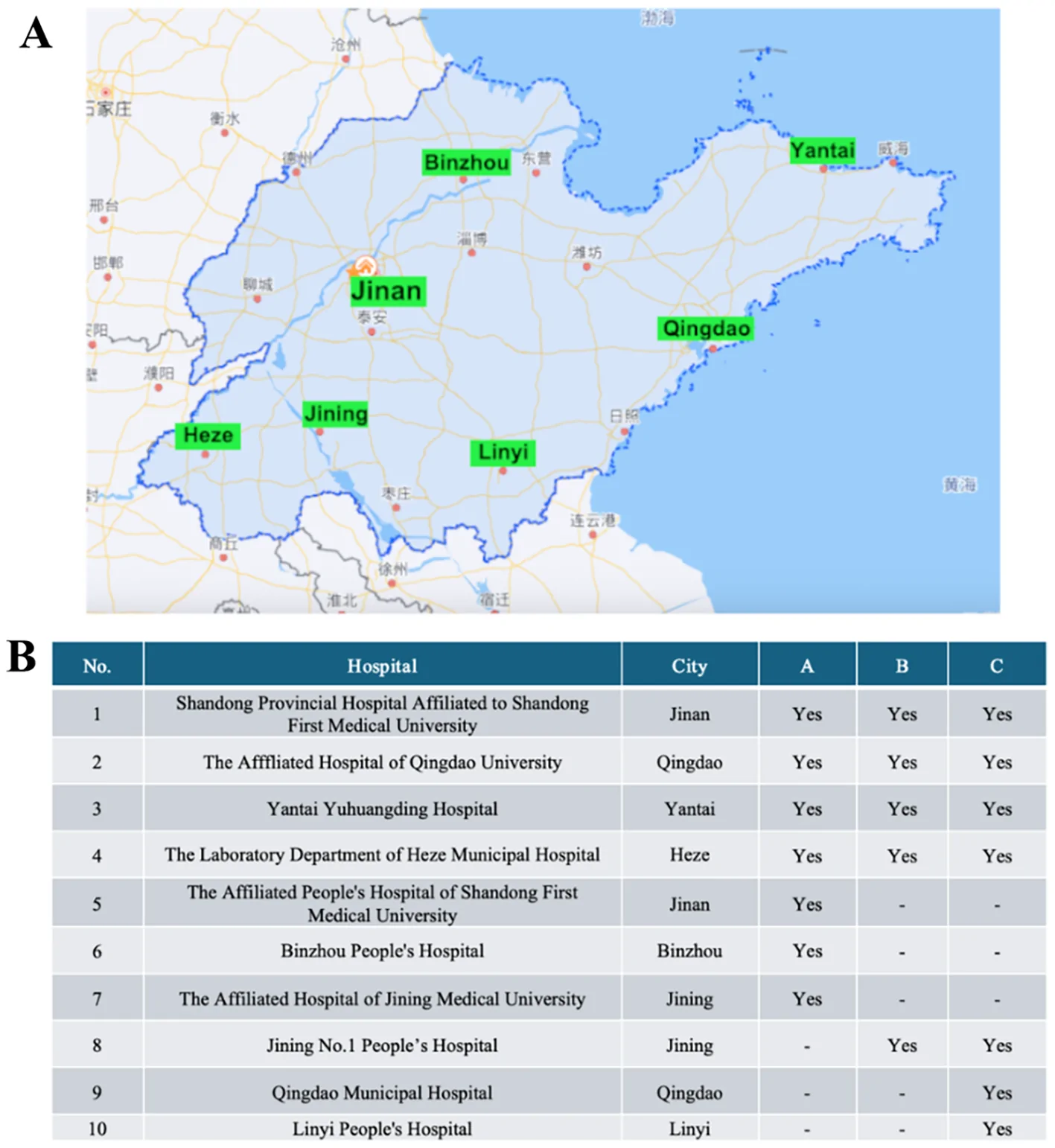
Geographic distribution and workflow optimization measures of participating hospitals **(A)** Geographic distribution of the 10 participating hospitals in Shandong Province, China. **(B)** Summary of workflow optimization implementation across participating hospitals. A: 24-hour uninterrupted blood culture specimen processing and loading; B: rapid MALDI-TOF MS ID at 6–8 h post-BC positivity and reporting to relevant medical staff with streamlined AST; C: next-day ID and reporting to relevant medical staff with streamlined AST.

### 2.5 Outcome measures

Primary Outcome

The primary study outcome was the change in key BC TAT metrics between the pre- and post-optimization phases, defined as:

TAT1: Time from blood collection to Gram-stain result reporting (hours)

TAT2: Time from blood collection to MALDI-TOF MS ID with result reporting (hours)

TAT3: Time from blood collection to final AST result reporting (hours)

Secondary Outcomes

Pathogen distribution: Isolation proportion of Gram-positive bacteria, Gram-negative bacteria and fungi in pre- vs. post-optimization cohorts, plus distribution of top isolated species.

AST reporting efficiency: Median AST TAT stratified by participating hospital and major BSI pathogens;

Clinical outcomes: All-cause in-hospital mortality, hospital length of stay (LOS, days), and total duration of systemic antibiotic administration (days).

Antimicrobial adjustment metrics: Median time from blood collection to initiation of targeted narrow-spectrum antibiotics (three time nodes: post-Gram-stain, post-MS ID, post-final AST); frequency of antibiotic addition and de-escalation within 72 h after final AST results;

Healthcare economic outcomes: Total hospitalization costs, laboratory testing costs and antimicrobial-related costs (unit: Chinese Yuan, ¥).

### 2.6 Statistical analysis

All statistical computations were performed using SPSS 26.0 (IBM, USA) and R 4.3.3 (R Foundation, Austria). Shapiro-Wilk test was applied to judge normality of continuous variables. Non-normal distributed data were described as median (interquartile range, IQR) and inter-group comparisons conducted via Mann-Whitney U test. Categorical variables were summarized as counts (percentages), compared with Pearson χ² test; Fisher’s exact test was adopted when theoretical frequency < 5. Two-tailed *P* < 0.05 was defined as statistically significant. This retrospective cohort study lacked pre-specified sample size calculation; all consecutive eligible patients during the study period were enrolled.

Given the obvious baseline imbalance between pre- and post-optimization cohorts in demographic and clinical characteristics, propensity score-based stabilized inverse probability treatment weighting (IPTW) was implemented to eliminate selection confounding and obtain unbiased estimates of optimization’s independent clinical and economic effects. Multivariate logistic regression was used to calculate propensity scores, with adjustment covariates including age, gender, admission department, fungal infection and sepsis diagnosis. Absolute standardized mean difference (SMD) was adopted to evaluate covariate balance after weighting; SMD < 0.10 was defined as adequate balance. The positivity assumption was validated through visual overlap of weighted sample distribution plots. All IPTW-related calculations were completed via the WeightIt R package. Detailed formula, calculation rules and parameter settings of IPTW are presented in **Supplementary Methods**.

As sensitivity analyses to verify the robustness of IPTW findings, multi-variable regression models were additionally constructed for all primary clinical and economic outcomes. Binary logistic regression was applied for all-cause in-hospital mortality, and linear regression models for hospital length of stay and all cost indicators. All regression models adjusted for age, gender, admission department, fungal infection, and sepsis diagnosis with participating hospital included as a fixed effect to account for inter-center cost heterogeneity and baseline practice differences. No medical service price adjustments were issued across the study period by provincial healthcare authorities, so inflation-related year-to-year cost drift was considered negligible. All adjusted regression results were directionally consistent with IPTW-weighted estimates, confirming the stability of the core findings.

## 3 Results

### 3.1 Baseline characteristics and covariate balance

A detailed patient screening flowchart illustrating the inclusion and exclusion process is provided in **Supplementary Figure 1**. A total of 1, 826 eligible adult patients were enrolled in this multicenter retrospective study, including 942 individuals in the pre-optimization cohort and 884 patients in the post-optimization cohort. Unadjusted raw baseline comparisons revealed prominent intergroup imbalances across multiple key clinical covariates. Specifically, the pre-optimization group contained a higher proportion of intensive care unit (ICU) admissions (36.4% vs. 28.8%), while the post-optimization group had more emergency intensive care unit (EICU) patients (7.2% vs. 3.9%) and female inpatients (43.9% vs. 38.1%). Full unweighted baseline demographic and clinical data are summarized in **Supplementary Table 8**.

IPTW based on propensity scores was applied to eliminate confounding bias from baseline variables. Following weighting, all absolute SMDs across measured covariates fell below 0.01, indicating excellent covariate balance between the two groups (**Table 1**). The positivity assumption required for valid IPTW analysis was fully satisfied, as visual inspection of propensity score weight distributions confirmed robust overlap without extreme weight values (**Supplementary Figure 2**).

**Table 1:** Baseline covariates after IPTW balancing.

Characteristics	Total (n=1, 824.6)	Pre-optimization group (n=939.8)	Post-optimization group (n=884.9)	SMD	*P*-value^a^
Age, (median [IQR]), years	66.0 [55.0, 74.0]	66.0 [54.0, 74.0]	66.0 [56.0, 74.0]	0.004	0.971
Gender					
Male, n (%)	744.2 (40.8)	338.3 (40.7)	361.3 (40.8)	0.002	0.973
Female, n (%)	1080.5(59.2)	556.8 (59.3)	523.6 (59.2)		
Admission department, n					
Internal medicine, n (%)	618.9 (33.9)	318.5 (33.9)	300.5 (34.0)	0.001	0.976
Surgery, n (%)	332.7 (18.2)	171.1 (18.2)	161.6 (18.3)	0.002	0.974
ICU, n (%)	601.7 (33.0)	310.1(33.0)	291.6 (33.0)	0.001	0.982
EICU, n (%)	101.3 (5.5)	52.3 (5.6)	49.0 (5.5)	0.002	0.975
Emergency, n (%)	100.3 (5.5)	52.4 (6.4)	47.9 (5.4)	0.007	0.886
Other, n (%)	69.7 (3.8)	35.3 (2.5)	34.4 (3.8)	0.004	0.907
Fungi infection, n (%)	22 (1.2)	17 (1.8)	5 (0.6)	0.115	0.027
Sepsis diagnosis, n (%)	778.9 (43.0)	400.7 (42.9)	378.2 (43.1)	0.004	0.931
Sepsis shock, n (%)	304.6(16.8)	163.6(17.5)	141.0(16.1)	0.039	0.418

a *P*-values for numerical variables were calculated using Wilcoxon rank sum test if not explicitly specified; *P*-values for categorical variables were calculated using Chi-square tests.

### 3.2 Pathogen distribution

A total of 1, 763 pathogenic isolates were recovered from the 1826 enrolled BSI episodes, with 916 isolates from the pre-optimization period and 847 from the post-optimization period (**Table 2**). Gram-negative organisms remained the dominant pathogen category in both cohorts, yet their isolation proportion rose after workflow implementation (72.0% vs. 79.7%). By contrast, the recovery rates of Gram-positive bacteria (26.1% vs. 19.7%), and fungi (1.8% vs. 0.5%) decreased in the post-optimization phase.

**Table 2:** Distribution of pathogens isolated from blood cultures in pre- and post-optimization groups.

Pathogen category/species	Pre-optimization group (n=916 isolates)	Post-optimization group (n=847 isolates)
Gram positive organisms	239 (2 6. 1 %)	167 (19.7 %)
CoNS	64 (7.0%)	32 (3.8%)
*Enterococcus* spp.	61(6.6%)	46 (5.4%)
*Staphylococcus aureus*	61 (6.6%)	48 (5.7%)
*Streptococcus* spp.	30 (3.3%)	22 (2.6%)
Gram negative organisms	660 (72.0 %)	675 (79. 7 %)
*Escherichia coli*	307 (33.5%)	352 (41.5%)
*Klebsiella* spp.	178 (19.4%)	164 (19.3%)
*Pseudomonas aeruginosa*	43 (4.7%)	26 (3.0%)
*Enterobacter* spp.	24 (2.6%)	19 (2.2%)
*Acinetobacter* spp.	37 (4.0%)	19 (2.2%)
Fungi	17 (1.8 %)	5 (0. 5 %)

The five most frequently isolated pathogens remained consistent across both study periods: *Escherichia coli, Klebsiella* spp.*, coagulase-negative staphylococci* (CoNS)*, Enterococcus* spp.*, and Staphylococcus aureus.* The temporal shift in pathogen distribution was interpreted as secular epidemiological fluctuation rather than an effect of laboratory workflow optimization, given that hospital infection control protocols and empirical antimicrobial regimens remained unchanged throughout the study window.

### 3.3 Impact of optimization on BC TAT metrics

All three core blood culture TAT endpoints were significantly shortened after the workflow intervention (**Table 3**). TAT1 decreased from 40.2 (IQR, 25.8–53.6) hours to 23.6 (IQR, 17.4–34.9) hours (*P* < 0.001). TAT2 fell from 43.8 (IQR, 37.0–65.1) hours to 33.0 (IQR, 27.3–44.0) hours (*P* < 0.001). TAT3 was reduced from 70.2 (IQR, 61.9–85.9) hours to 52.2 (IQR, 44.0–68.3) hours (P < 0.001). Collectively, the workflow optimization substantially accelerated the full diagnostic process from blood collection to complete susceptibility reporting.

**Table 3:** BC TAT metrics in pre- and post-optimization groups.

TAT metric, median [IQR], hours	Total (n=1826)	Pre-optimization group (n=942)	Post-optimization group (n=884)	*P*-value^a^
TAT_1_	26.9 [21.2, 41.0]	37.0 [25.0, 49.4]	23.6 [17.9, 33.9]	<0.001
TAT_2_	34.3 [29.1, 43.8]	43.9 [36.9, 65.6]	32.9 [28.2, 40.9]	<0.001
TAT_3_	65.0 [49.5, 76.5]	70.4 [63.4, 84.9]	51.9 [44.5, 67.3]	<0.001

a Mann- Whitney U test.

### 3.4 Inter-hospital variability and dose–response of AST reporting efficiency

Substantial inter-hospital heterogeneity in AST TAT reduction was observed across the 10 participating tertiary centers (**Figure 2A**). 9 out of 10 hospitals achieved a median AST TAT reduction ranging from 6.2 to 25.1 hours, whereas Hospital No.9 exhibited no measurable shortening of reporting lag.

**Figure 2:**
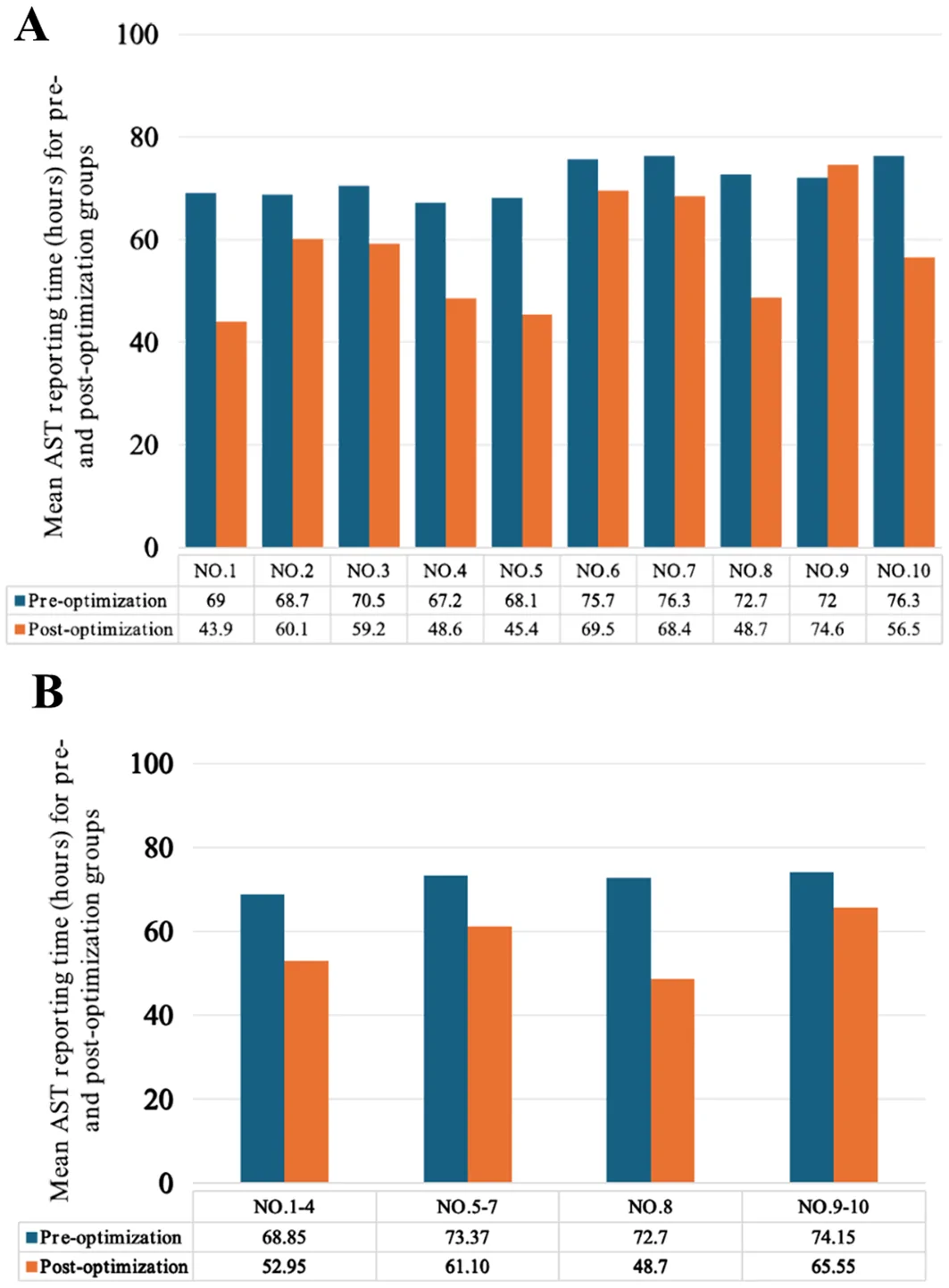
AST reporting time before and after laboratory workflow optimization. **(A)** Median AST TAT in the 10 participating tertiary hospitals in Shandong Province, China, comparing pre-optimization (blue) and post-optimization (orange) phases. Nine of the 10 centers showed measurable reductions in AST TAT, ranging from 6.2 to 25.1 hours. Data are presented as median values in hours. All between-group comparisons were performed using the Mann–Whitney U test (two-tailed). **(B)** The 10 hospitals were stratified into four subgroups according to the optimization modules adopted.

To quantify the incremental benefit of individual intervention components, the 10 hospitals were stratified into four subgroups according to the optimization modules adopted (**Figure 2B**). A clear dose–response relationship was observed: hospitals implementing all three components achieved the largest AST TAT reduction, while sites with fewer intervention modules showed progressively smaller improvements. Hospital 8, which adopted dual MALDI-TOF ID without 24/7 reception, exhibited the largest absolute TAT reduction, likely attributable to its longer baseline diagnostic lag and smaller sample volume. Detailed subgroup AST TAT data for all four implementation patterns are provided in **Supplementary Table 10**.

When stratified by pathogen taxonomy, median AST TAT was significantly shortened for all major bloodstream pathogens following workflow adjustment (**Figure 3**). For main Gram-negative bacilli, TAT reductions ranged from 17.1 to 22.1 hours, with Pseudomonas aeruginosa showing the maximum 22.1-hour decrease. For main Gram-positive organisms, AST turnaround was cut by 19–19.7 hours.

**Figure 3:**
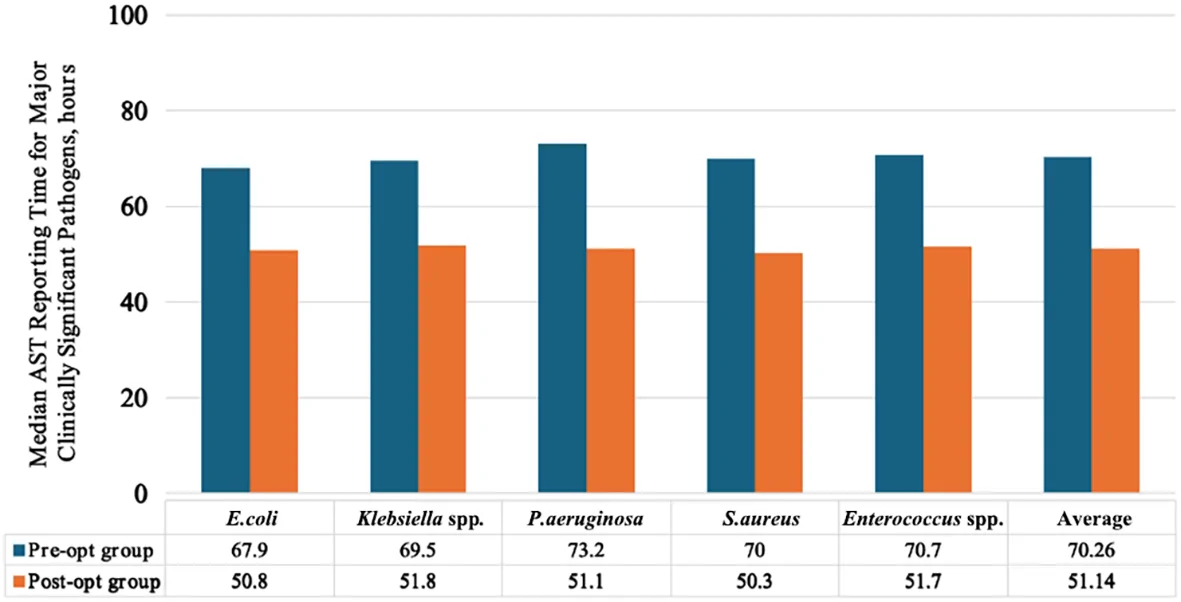
Pathogen-stratified AST reporting time before and after optimization. Median AST TAT for major clinically significant bloodstream pathogens, comparing pre-optimization (Pre-opt) and post-optimization (Post-opt) phases. All listed pathogens showed statistically significant reductions in AST TAT after workflow implementation. For Gram-negative bacilli, TAT reductions ranged from 17.1 to 22.1 hours, with *Pseudomonas aeruginosa* showing the largest improvement; for Gram-positive organisms, AST TAT was shortened by 19.0–19.7 hours. Data are presented as median values in hours. Statistical comparisons were performed using the Mann–Whitney U test (two-tailed).

### 3.5 Impact of optimization on antibiotic use metrics

Two main antimicrobial adjustment endpoints were analyzed in this study. Addition was defined as starting new antibiotics based on culture and susceptibility results. Elimination, or de-escalation, referred to stopping unnecessary agents or narrowing the spectrum once definitive AST results were available. While transitioning to pathogen-directed targeted therapy is the underlying clinical goal, this study quantified only the frequency of additions and de-escalations.

After workflow implementation, the median interval from blood culture collection to initiation of targeted antimicrobial therapy was significantly shortened across all three diagnostic timepoints (all *P* < 0.001, **Table 4**): The median time from blood collection to targeted therapy was reduced from 68.1 h to 48.6 h after Gram-stain reporting, from 86.5 h to 62.1 h after MS ID result reporting, and from 96.4 h to 77.6 h after final AST result reporting.

**Table 4:** Comparison of patient groups based on the time of changes in antibiotic therapy.

Time variable (Time from blood collection to targeted antibiotic therapy with in 72h), median [IQR], hours	Pre-optimization group	Post-optimization group	*P*-value
after gram staining reporting	68.1 [47.7, 91.0]	48.6 [30.0, 70.5]	<0.001
after MS identification result reporting	86.5 [68.4, 107.1]	62.1 [46.5, 80.2]	<0.001
after final AST result reporting	96.4 [77.4, 116.9]	77.6 [59.8, 101.2]	<0.001
Addition^a^ (n)	344	279	<0.001
Elimination^b^ (n)	407	321	<0.001

a Addition: the initiation of any new antibiotic therapy.

b Elimination: the discontinuation of antibiotic therapy or a reduction in the number or spectrum of administered antibiotics.

In parallel, the frequency of antimicrobial regimen revisions within 72 hours of final AST results declined significantly in the post-optimization cohort (all *P* < 0.001). The number of patients requiring additional antimicrobial agents fell from 344 to 279, while instances of therapy de-escalation or discontinuation decreased from 407 to 321.

### 3.6 IPTW-adjustment clinical and economic outcomes

After adjusting for baseline confounders using IPTW, the post-optimization group demonstrated significantly better outcomes compared to the pre-optimization group across multiple measures.

For clinical endpoints, all-cause in-hospital mortality was markedly lower in the post-optimization weighted cohort (7.5% vs. 12.4%). No meaningful intergroup difference was detected in the median duration of systemic antibiotic therapy and LOS (**Table 5**).

**Table 5:** Clinical outcomes After IPTW adjustment.

Outcome	Total (n=1, 824.6)	Pre-optimization group (n=939.8)	Post-optimization group (n=884.9)	*P*-value^a^
Hospital Mortality, n (%)	152.7 (10.0)	95.7 (12.4)	57.0 (7.5)	0.002
Antibiotics usage time, day (median [IQR])	13.0 [8.0, 21.0]	13.0 [8.0, 21.0]	13.0 [9.0, 20.0]	0.965
LOS, (median [IQR])	15.0 [9.0, 25.1]	15.0 [9.0, 26.0]	15.0 [9.0, 25.0]	0.179

a *P*-values for continuous variables were calculated via design-based Wald tests; categorical variables were analyzed using Rao-Scott adjusted chi-square tests.

Percentages for hospital mortality were calculated among patients with non-missing in-hospital outcome data. Fractional counts result from IPTW weighting.

Substantial reductions in core economic indicators were observed following workflow adjustment. The weighted mean total hospitalization cost was ¥104, 537.2 in the post-optimization group, compared with ¥135, 429.6 in the pre-optimization group. Laboratory testing costs showed no statistically significant intergroup disparity after IPTW adjustment. The expenditure on antibacterial drugs shows a downward trend: weighted mean antibiotic costs dropped from ¥23, 867.2 to ¥12, 126.7, representing a nearly 50% cost reduction for antibiotics. Non-significant secondary outcome metrics including sepsis shock prevalence and laboratory costs are summarized in **Table 6**. These cost findings should be interpreted cautiously given the retrospective observational design and potential residual confounding from unmeasured factors such as detailed comorbidity burden, prior antimicrobial exposure, and appropriateness of initial empiric therapy. Sensitivity analyses using multivariable regression models yielded directionally consistent results with IPTW-weighted estimates for all clinical and economic outcomes (**Supplementary Tables 1**–**7**), Both methods demonstrated that workflow optimization significantly lowered hospital mortality, hospital total cost and antibiotics cost, confirming the robustness of the core findings.

**Table 6:** Economic outcomes after IPTW adjustment (Unit: Chinese Yuan, ¥).

Category	Total (n=1, 824.6)	Pre-optimization group (n=939.8)	Post-optimization group (n=884.9)	*P*-value^a^
Total hospital costs (mean (SD))^b^	120811.1 (218011.9)	135429.6 (254319.6)	104537.2 (167298.2)	0.003
Laboratory cost (mean (SD))^c^	39875.0 (888093.8)	63844.7 (1223966.2)	13252.8 (17884.0)	0.282
Antibiotics cost (mean (SD))	18280.6 (54941.1)	23867.2 (67411.5)	12126.7 (35630.7)	<0.001

^a^*P*-values for continuous variables were calculated via design-based Wald tests; categorical variables were analyzed using Rao-Scott adjusted chi-square tests.

^b^Total hospital costs covered general treatment fees, imaging expenses, surgical charges, medication, rehabilitation and medical consumables.

^c^Laboratory costs included cytological, microbiological, immunological, biochemical and molecular testing fees.

## 4 Discussion

This multicenter retrospective study evaluated the clinical and economic impact of a three-component blood culture workflow optimization across 10 tertiary hospitals in Shandong Province. To our knowledge, this is the first real-world multicenter analysis to quantify the incremental TAT gains from different intervention combinations, and to confirm associated changes in clinical outcomes and costs after IPTW adjustment for baseline confounding. In line with earlier single-center reports on partial laboratory improvements (Ai et al., 2024; Dai et al., 2024; Qin et al., 2025), the full optimization package shortened all key diagnostic intervals, and cut antimicrobial expenditure substantially, though hospital length of stay remained unchanged—without increasing mortality or prolonging overall antibiotic exposure. These findings offer a practical, scalable blueprint for clinical microbiology laboratories in tiered healthcare systems.

The 24/7 specimen reception model eliminated the overnight backlog inherent to daytime-only workflows. Specimens collected after hours were previously held at room temperature or 4 °C until the next shift, delaying incubation and potentially reducing pathogen recovery (Weinstein et al., 1997). Immediate loading upon arrival accelerates the entire diagnostic chain from the pre-analytical stage, which is particularly relevant given that ICU, EICU and emergency patients accounted for 44.0% of the cohort.

The 6–8 h short-incubation MALDI-TOF ID bypassed the ≥72 h pure subculture step that traditionally represents the major workflow bottleneck (Zengin Canalp and Bayraktar, 2021; Padhy et al., 2024). Our findings are consistent with published validation data showing 99.7% species-level concordance for this approach (Demir and Hazirolan, 2024; Fitts et al., 2024). Gram-negative organisms, which predominated in our cohort, typically yield sufficient biomass within 6–8 hours; Gram-positive and fastidious species that grow more slowly are covered by the supplementary next-day ID module, balancing speed and diagnostic reliability.

Finally, initiating AST immediately after MALDI-TOF ID compressed the post-positivity interval by 24–48 hours compared with the conventional practice of waiting for pure colony isolation. Because final AST results guide definitive targeted therapy, any delay in set-up directly postpones optimal antibiotic selection (Briggs et al., 2021; Fabre et al., 2022). The overall 18.5-hour reduction in AST TAT we observed translates directly into earlier opportunities for treatment adjustment.

A notable finding is the graded improvement in AST TAT according to how many optimization components each hospital adopted. Sites implementing all three modules achieved the largest reduction (15.9 h on average), whereas those with only a single intervention showed more modest gains (8.6 h). Hospital 8, which used both rapid and next-day ID without 24/7 reception, recorded the largest absolute improvement (24 h), likely reflecting its longer baseline TAT. Hospital 9, with next-day ID only, showed no measurable AST gain, reinforcing that isolated changes cannot fully address multi-step workflow delays. This stepwise pattern helps laboratories with limited staffing or space to prioritize interventions according to local capacity.

The economic finding was the roughly 50% reduction in weighted antimicrobial costs after IPTW adjustment. Two mechanisms likely underlie the observed cost reduction. First, earlier pathogen and susceptibility results enabled earlier transition to narrow-spectrum targeted therapy, reducing the duration of expensive broad-spectrum empiric antibiotic use. Second, both additions and de-escalations within 72 hours of final AST reporting decreased after optimization, indicating more accurate initial regimen selection and fewer unnecessary treatment adjustments—both of which contribute to lower overall antimicrobial expenditure. LOS was comparable between the two weighted groups, suggesting that faster microbiological diagnosis alone may not be sufficient to shorten inpatient stay in tertiary centers where multiple factors beyond antimicrobial timing influence discharge decisions. It should be noted that these economic findings are derived from an observational before-after design and may be influenced by unmeasured confounders. Future prospective studies with more comprehensive covariate adjustment are needed to confirm these cost findings.

After IPTW adjustment, in-hospital mortality was significantly lower in the post-optimization group (7.5% vs. 12.4%, P = 0.002), though the absolute reduction was modest at 4.9 percentage points and should be interpreted cautiously given the observational design. We suspect this relatively small effect reflects the high baseline standard of care across these tertiary centers: empiric broad-spectrum antibiotics were already started promptly after Gram stain reporting before optimization, so further TAT gains offer limited additional mortality benefit. This is consistent with earlier single-center work (Dai et al., 2024; Qin et al., 2025) and suggests mortality improvements may be larger in lower-resource settings. Total antibiotic duration was unchanged, indicating faster diagnosis optimized treatment type rather than overall duration.

The shift in pathogen distribution—rising Gram-negatives, falling Gram-positives and fungi—most likely reflects secular epidemiological trends rather than an effect of workflow optimization. Blood collection standards, infection control practices, and empiric antibiotic guidelines all remained stable across the study period, and the top five species were unchanged. This supports the interpretation that clinical and economic improvements were driven by the laboratory intervention itself, consistent with year-to-year variation commonly seen in retrospective infectious disease cohorts (Kariv et al., 2013; Qin et al., 2025).

Key strengths include the multicenter real-world design across seven cities in Shandong Province, the large sample size (1, 826 BSI episodes), the integrated three-component intervention, uniform instrumentation across sites, multi-dimensional outcome assessment, and IPTW adjustment to reduce confounding from retrospective grouping.

Several limitations should be noted. A sensitivity analysis excluding the COVID-19 peak quarter (Q4 2022) produced nearly identical results to the full cohort, suggesting minimal confounding from pandemic-related disruption. Nevertheless, unmeasured residual confounding cannot be ruled out—we lacked data on formal severity scores, detailed comorbidity burden, and prior antibiotic exposure. We did not assess the appropriateness of initial empiric antimicrobial therapy or detailed prior antibiotic exposure history, which may contribute to residual confounding in the cost analysis. Generalizability is limited to tertiary hospitals in Shandong and may not extend to primary care, grassroots facilities, or regions with different antimicrobial stewardship structures. Only in-hospital outcomes were captured; 30-day or 90-day mortality and long-term resistance effects remain unknown. We also did not measure rates of inappropriate initial empiric therapy, an important metric of diagnostic stewardship impact.

## 5 Conclusions

In summary, this multicenter retrospective IPTW-adjusted analysis demonstrates that the optimization strategy combining 24/7 uninterrupted specimen processing, 6–8 h short-incubation rapid MALDI-TOF MS ID, and second-day microbial ID markedly shortens all core BC diagnostic timelines, generates graded AST efficiency gains proportional to the number of adopted intervention components and cuts nearly half of antimicrobial expenditure without altering overall antibiotic exposure, LOS or in-hospital mortality. Our standardized three-module workflow provides a low-cost, operable optimization blueprint for clinical microbiology laboratories in tertiary hospitals. Future large-scale prospective multi-region trials with long-term patient follow-up are warranted to further validate whether this rapid diagnostic strategy reduces long-term mortality and curbs regional antimicrobial resistance development.

## Data Availability

The raw data supporting the conclusions of this article will be made available by the authors, without undue reservation.
