# Supplementary material for: The impact of multicenter microbiology laboratory workflow optimization on antimicrobial susceptibility testing reporting time and outcomes of patients with bloodstream infection
Source: Front Cell Infect Microbiol. 2026 Sep 16;16:1875923. doi: 10.3389/fcimb.2026.1875923 (PMC13623717; doi:10.3389/fcimb.2026.1875923)
Supplement: Supplementary file 1 [file DataSheet1.pdf]

Supplementary Material

1 Supplementary Data

Supplementary Material should be uploaded separately on submission. Please include any supplementary data, figures and/or tables.

Supplementary material is not typeset so please ensure that all information is clearly presented, the appropriate caption is included in the file and not in the manuscript, and that the style conforms to the rest of the article.

1.1 BSI Diagnostic Criteria and Contamination Adjudication

Diagnostic Standards

All BSI cases were diagnosed according to the combined criteria of the CLSI guidelines and CDC NHSN laboratory-confirmed bloodstream infection (LCBI) criteria. A confirmed BSI required both positive blood culture results and clinical manifestations of infection (fever, chills, hypotension, leukocytosis or leukopenia).

Recognized Pathogens (Single-Set Positive Confirms BSI)

Isolation of any of the following recognized pathogenic microorganisms from a single blood culture set was sufficient to confirm true BSI:

Staphylococcus aureus

Streptococcus pneumoniae

β-hemolytic streptococci

Enterococcus spp.

Escherichia coli and other members of the *Enterobacterales*

Pseudomonas aeruginosa

Listeria monocytogenes

Neisseria meningitidis

Haemophilus influenzae

Anaerobic Gram-negative rods (Bacteroides spp*., Fusobacterium* spp*.)*

Candida spp.

Staphylococcus lugdunensis, despite being a *coagulase-negative staphylococcus* (CoNS), was classified as a recognized pathogen due to its high virulence and clinical significance[19].

Skin Commensals (Multi-Set Positive Required)

For common skin commensal contaminants (CoNS, *Cutibacterium acnes, corynebacteria, Bacillus* spp.*, viridans group streptococci, Micrococcus* spp.*, Aerococcus* spp.*, Lactobacillus* spp.), isolation from ≥2 separate culture sets collected within 48 hours, accompanied by infectious clinical symptoms, was required to diagnose true BSI[1].

Three-Grade Contamination Adjudication System

A graded judgment framework was established to distinguish true BSI from specimen contamination:

Definite contamination: Single blood culture set positive, time-to-positivity > 36 h, no infectious clinical manifestations, no indwelling intravascular catheters — excluded directly.

Probable contamination: Single set positive accompanied by 1–2 contamination risk factors — excluded after infectious diseases physician review.

Definite clinically significant BSI: ≥2 independent venipuncture culture sets positive; or single set positive matching catheter tip isolate with consistent species; or single set positive with clear clinical signs and targeted anti-infective treatment initiated by clinicians — formally enrolled.

Additional supportive criteria for contamination included time-to-positivity ≥ 48 hours and discordant growth pattern (positive in only one bottle of a two-bottle set).

1.2 Detailed Statistical Methods

Propensity Score Estimation

Propensity scores (PS) were estimated using multivariate logistic regression, in which treatment assignment (pre-optimization vs. post-optimization) was modeled as a function of pre-specified baseline covariates considered potential confounders of the treatment-outcome relationship.The propensity score model included the following baseline variables:

- Gender
- Age
- Admission department (internal medicine, surgery, ICU, EICU, emergency, others)
- Fungal infection
- Sepsis diagnosis

Inverse Probability Treatment Weighting

Stabilized inverse probability treatment weights were calculated based on propensity scores as:

$$sw_i = \begin{cases} \dfrac{P(T = 1)}{PS_i}, & \text{if treated} \\ \dfrac{P(T = 0)}{1 - PS_i}, & \text{if untreated} \end{cases}$$

where sw_i denotes the stabilized weight for subject i, PS_i denotes the estimated propensity score for subject i, and $P(T = 1)$ and $P(T = 0)$ represent the proportions of patients in the treatment and control groups, respectively.

Positivity Assumption Verification

As the positivity assumption is a prerequisite for valid IPTW adjustment, we verified this assumption by visually inspecting the overlap of weight distributions between the two patient groups. Good overlap with no extreme weights was considered evidence that the positivity assumption was satisfied.

Covariate Balance Assessment

Absolute standardized mean differences (SMDs) were calculated for all covariates before and after weighting to assess covariate balance.

For continuous variables, SMDs were defined as the difference in group means divided by the pooled standard deviation.

For binary variables, SMDs were calculated using pooled proportions.

For multicategory variables, balance diagnostics were assessed at the level of individual indicator variables corresponding to each category.

Adequate covariate balance was defined a priori as an absolute SMD of less than 0.10. Although p-values were reported descriptively, balance assessment primarily relied on SMDs rather than statistical significance tests.

Variable Presentation

Baseline characteristics and outcome variables were summarized before and after IPTW adjustment:

Continuous variables: reported as mean (standard deviation) for approximately normally distributed data, and as median (interquartile range) for non-normally distributed data.

Categorical variables: reported as counts (percentages).

For multicategory variables, the proportion of participants within each category level was presented separately.

Statistical Software and Tests

All analyses were performed using R software (R version 4.3.3; R Core Team, 2024. R: A Language and Environment for Statistical Computing. R Foundation for Statistical Computing, Vienna, Austria. https://www.R-project.org/).The WeightIt package (version 1.3.0; Greifer N, 2024. WeightIt: Weighting for Covariate Balance in Observational Studies. https://CRAN.R-project.org/package=WeightIt) was used for IPTW analysis.

For weighted analyses:

P-values for numerical variables were calculated using design-based Wald tests

P-values for categorical variables were calculated using Rao-Scott adjusted chi-square tests

For unweighted analyses:

P-values for age were calculated using Wilcoxon rank sum test

P-values for other continuous variables were calculated using t-test

P-values for categorical variables were calculated using Chi-squared test

1.3 Sensitivity analysis: multi-variate regression analysis for outcome characteristics

The following baseline variables considered potential confounders of the treatment-outcome relationship was used for multi-variate regression analysis.

- Gender
- Age
- Admission department
- Fungi infection
- Sepsis diagnosis

The following patient outcome characteristics were analyzed separately by multi-variate regression:

- Sepsis shock (binary, occurrence or no-occurrence)
- In hospital mortality (binary, dead or alive)
- Length of stay in hospital (continuous)
- Total hospital cost (continuous)
- Laboratory cost (continuous)
- Antibiotics cost (continuous)
- Antibiotics usage time (continuous)

For binary outcomes, logistic regression models were fitted using generalized linear models with a logit link and robust variance estimation. Effect estimates were reported as odds ratios (ORs) with corresponding 95% confidence intervals (CIs).

For continuous outcomes, multi-variate linear regression models were fitted. Effect estimates were reported as regression coefficient (β) with corresponding 95% confidence intervals (CIs).

Binary outcomes of sepsis shock

Logistic regression was used to investigate association between sepsis shock and workflow optimization. As predictor sepsis diagnosis demonstrated complete outcome separation for sepsis shock : all subjects positive for outcome of sepsis shock occurred in patients who were positive for sepsis diagnosis, while none of the sepsis diagnosis negative subjects presented the event of sepsis shock. Therefore, predictor sepsis diagnosis was excluded from the multivariable logistic regression.

Table 1 showed logistic regression results for outcome of sepsis shock. Compared to patients in pre-optimization group, patients in post-optimization group had 14% percent decrease in odds of sepsis shock (OR = 0.86). This increase did not reach statistical significance ($P = 0.3$).

Binary outcome of hospital mortality

Table 2 showed that patients in post-optimization group had lower in-hospital mortality. Compared to patients in pre-optimization group, patients in post-optimization group had 47% percent decrease in odds of mortality ($P < 0.001$).

Continuous outcome of length of stay in hospital

Table 3 showed regression results for outcome of length of stay in hospital. The results showed that patients in post-optimization group stayed slightly shorter (average 1.2 days) than patients in pre-optimization group. However, this difference did not show statistical significance ($P = 0.3$).

Continuous outcome of total hospital cost

Table 4 showed regression results for outcome of hospital cost. Patients in post-optimization group cost significantly much less (32789 RMB less, $P < 0.001$) than patients in pre-optimization.

Continuous outcome of laboratory cost

Table 5 listed regression results for outcome of laboratory cost. The results showed that patients in post-optimization group cost fewer (49714 RMB less) than patients in pre-optimization group. However, this difference was not statistically significant ($P = 0.2$). It should be noted that one patient in pre-optimization group had overwhelmingly greater lab cost (lab cost of 31853959.6 RMB during stay in hospital) than other patients. This patient was identified as an outlier in statistical diagnosis of linear regression. When this patient was excluded from linear regression analysis, the sign of the coefficient for optimization intervention remained unchanged. However, due to decrease of standard error of the coefficient, the coefficient appeared to be statistically significant after excluding this patient from analysis (data not shown).

Continuous outcome of antibiotics cost

Table 6 showed regression results for outcome of antibiotics cost. The results showed that antibiotics cost for patient in post-optimization group was significantly fewer than those in pre-optimization (12302 RMB fewer, $P < 0.001$).

Continuous outcome of length of antibiotics usage time

Table 7 showed regression results for outcome of antibiotics usage time. The results showed that there was no significant difference for antibiotics usage time between two patient groups.

Conclusion

Our results showed that regression analysis results qualitatively agree with analysis results based on IPTW method. Both methods demonstrated that workflow optimization significantly lowered hospital mortality, hospital total cost and antibiotics cost.

Supplementary Table 1. Multi-variate logistic regression model for sepsis shock

Characteristic	OR	95% CI	*P*-value
**Gender**			
Female	—	—	
Male	1.12	0.85, 1.48	0.4
**Treat intervention**			
Pre-optimization	—	—	
Post-optimization	0.86	0.66, 1.13	0.3
**Age, years**	1.01	1.00, 1.02	0.043
**Admission department**			
Internal medicine	—	—	
Surgery	1.74	1.01, 2.98	0.045
ICU	10.5	7.11, 16.0	<0.001
EICU	6.29	3.48, 11.3	<0.001
Emergency	3.22	1.60, 6.24	<0.001
Others	0.00		>0.9
**Fungi**			
No	—	—	
Yes	1.44	0.48, 3.87	0.5

Abbreviations: CI = Confidence Interval, OR = Odds Ratio

Supplementary Table 2. Multi-variate logistic regression model for hospital mortality

Characteristic	OR	95% CI	*P*-value
**Gender**			
Female	—	—	
Male	1.35	0.94, 1.98	0.11
**Treat intervention**			
Pre-optimization	—	—	
Post-optimization	0.53	0.36, 0.75	<0.001
**Age, years**	1.02	1.01, 1.03	0.006
**Admission department**			
Internal medicine	—	—	
Surgery	1.29	0.65, 2.49	0.4
ICU	3.02	1.85, 5.09	<0.001
EICU	7.57	3.91, 14.7	<0.001
Emergency	2.31	1.0, 5.02	0.041
Others	0.00		>0.9
**Fungi**			
No	—	—	
Yes	2.31	0.61, 7.19	0.2
**Sepsis**			
No	—	—	
Yes	2.51	1.72, 3.68	<0.001

Abbreviations: CI = Confidence Interval, OR = Odds Ratio

Supplementary Table 3. Multi-variate linear regression model for length of stay in hospital

Characteristic	Beta	95% CI	*P*-value
**Gender**			
Female	—	—	
Male	1.7	-0.76, 4.2	0.2
**Treat intervention**			
Pre-optimization	—	—	
Post-optimization	-1.2	-3.6, 1.2	0.3
**Age, years**	0.03	-0.05, 0.12	0.4
**Admission department**			
Internal medicine	—	—	
Surgery	7.9	4.5, 11	<0.001
ICU	12	9.4, 15	<0.001
EICU	11	5.3, 16	<0.001
Emergency	12	6.1, 17	<0.001
Others	-3.6	-22, 15	0.7
**Fungi**			
No	—	—	
Yes	7.7	-3.0, 18	0.2
**Sepsis**			
No	—	—	
Yes	-3.5	-6.1, -0.99	0.006

Abbreviation: CI = Confidence Interval

Supplementary Table 4. Multi-variate linear regression model for total hospital cost

Characteristic	Beta	95% CI	*P*-value
**Gender**			
Female	—	—	
Male	37,328	17,357, 57,299	<0.001
**Treat intervention**			
Pre-optimization	—	—	
Post-optimization	-32,789	-51,995, -13,584	<0.001
**Age, years**	-1,316	-1,995, -637	<0.001
**Admission department**			
Internal medicine	—	—	
Surgery	106,567	78,943, 134,191	<0.001
ICU	155,131	130,978, 179,283	<0.001
EICU	64,231	18,793, 109,669	0.006
Emergency	37,131	-8,622, 82,884	0.11
Others	-23,945	-187,874, 139,984	0.8
**Fungi**			
No	—	—	
Yes	108,522	22,637, 194,408	0.013
**Sepsis**			
No	—	—	
Yes	28,535	7,914, 49,157	0.007

Abbreviation: CI = Confidence Interval

Supplementary Table 5. Multi-variate linear regression model for laboratory cost

Characteristic	Beta	95% CI	*P*-value
**Gender**			
Female	—	—	
Male	420	-73,647, 74,486	>0.9
**Treat intervention**			
Pre-optimization	—	—	
Post-optimization	-49,714	-120,930, 21,502	0.2
**Age, years**	256	-2,262, 2,774	0.8
**Admission department**			
Internal medicine	—	—	
Surgery	9,985	-92,468, 112,438	0.8
ICU	21,038	-68,500, 110,576	0.6
EICU	15,720	-152,803, 184,242	0.9
Emergency	-3,953	-173,645, 165,738	>0.9
Others	21,919	-586,074, 629,912	>0.9
**Fungi**			
No	—	—	
Yes	6,410	-312,131, 324,951	>0.9
**Sepsis**			
No	—	—	
Yes	-3,274	-79,728, 73,181	>0.9

Abbreviation: CI = Confidence Interval

Supplementary Table 6. Multi-variate linear regression model for antibiotics cost

**Characteristic**	**Beta**	**95% CI**	***P*-value**
**Gender**			
Female	—	—	
Male	7,674	1,663, 13,684	**0.012**
**Treat intervention**			
Pre-optimization	—	—	
Post-optimization	-12,302	-18,049, -6,555	**<0.001**
**Age, years**	-257	-460, -55	**0.013**
**Admission department**			
Internal medicine	—	—	
Surgery	14,501	6,375, 22,627	**<0.001**
ICU	23,035	15,782, 30,287	**<0.001**
EICU	5,987	-8,503, 20,477	0.4
Emergency	5,328	-9,461, 20,116	0.5
Others	-3,229	-51,813, 45,354	0.9
**Fungi**			
No	—	—	
Yes	8,971	-16,708, 34,651	0.5
**Sepsis**			
No	—	—	
Yes	9,025	2,780, 15,270	**0.005**

Abbreviation: CI = Confidence Interval

Supplementary Table 7. Multi-variate linear regression model for antibiotics usage time

**Characteristic**	**Beta**	**95% CI**	***P*-value**
**Gender**			
Female	—	—	
Male	3.3	1.1, 5.4	0.003
**Treat intervention**			
Pre-optimization	—	—	
Post-optimization	-1.3	-3.4, 0.74	0.2
**Age, years**	0.08	0.01, 0.16	0.030
**Admission department**			
Internal medicine	—	—	
Surgery	3.5	0.54, 6.4	0.020
ICU	9.6	7.0, 12	<0.001
EICU	6.6	1.7, 11	0.009
Emergency	6.9	1.9, 12	0.007
Others	3.5	-13, 20	0.7
**Fungi**			
No	—	—	
Yes	25	16, 34	<0.001
**Sepsis**			
No	—	—	
Yes	-1.1	-3.4, 1.1	0.3

Abbreviation: CI = Confidence Interval

2 Supplementary Figures and Tables

For more information on Supplementary Material and for details on the different file types accepted, please see here.

Supplementary Table 8. Baseline covariates before IPTW balancing

Characteristics	Total (n=1,826)	Pre-optimization (n=942)	Post-optimization (n=884)	SMD	*P*-value[a]
**Age,** (median [IQR])，years	66.0 [55.0, 74.0]	66.0 [54.0, 74.0]	66.0 [56.0, 74.0]	0.024	0.750
**Gender**					
Male, n (%)	747 (40.9%)	359 (38.1%)	388 (43.9%)	0.118	0.014
Female, n (%)	1079 (59.1%)	583 (61.9%)	496 (56.1%)		
**Admission Department**					
Internal medicine, n (%)	620 (34.0)	321 (34.1)	299 (33.8)	0.005	0.948
Surgery, n (%)	337 (18.5)	157 (16.7)	190 (20.4)	0.095	0.0488
ICU, n (%)	598 (32.7)	343 (36.4)	255 (28.8)	0.162	0.001
EICU, n (%)	101 (5.5)	37 (3.9)	64 (7.2)	0.145	0.003
Emergency, n (%)	99 (5.4)	60 (6.4)	39 (4.4)	0.087	0.081
Other, n (%)	71 (3.9)	24 (2.5)	47 (5.3)	0.114	0.053
**Fungi**, n (%)	22 (1.2)	17 (1.8)	5 (0.6)	0.115	0.027
**Sepsis diagnosis**, n (%)	777 (43.0)	414 (44.1)	363 (41.7)	0.048	0.333

a p-values for age were calculated using Wilcoxon rank sum test; p-values for categorical variables were calculated using Chi-squared test.

Supplementary Table 9. Concordance between 6–8 h rapid MALDI-TOF MS identification and second-day conventional pure colony identification

**Indicator**	**Value** , n (%)
Total specimens processed with 6–8 h rapid MALDI-TOF MS	958 (100.0)
Species-level concordance with second-day conventional ID	954 (99.6)
Genus-level concordance with second-day conventional ID	956 (99.8)
Identification failure (insufficient early bacterial growth)	11 (1.1)
Mixed cultures (≥2 morphologically distinct organisms)	30 (3.1)
Species-level discordant isolates	4 (0.4)
Categories of discordant isolates	Fastidious Gram-positive cocci, rare non-fermenters

Supplementary Table 10. AST TAT stratified by optimization implementation subgroup

**Subgroup**	**Hospitals**	**Absolute Reduction (h)**	**Sample Size (Pre / Post)**
Full bundle (24/7 Reception + Rapid ID + Second-day ID)	1, 2, 3, 4	15.90	445 / 408
24/7 Reception only	5, 6, 7	12.27	260 / 195
Dual ID modules (Rapid ID + Second-day ID)	8	24.00	83 / 75
Second-day ID only	9, 10	8.60	156 / 169

AST TAT values represent median time from blood collection to final antimicrobial susceptibility testing result reporting (TAT_3) in hours. Subgroups were defined by the combination of optimization components implemented at each site. The full bundle subgroup achieved the largest TAT reduction among the three multi-site subgroups, demonstrating a clear dose-response relationship between intervention completeness and diagnostic efficiency improvement. Hospital 8 (dual ID modules) showed the greatest absolute reduction, partly due to its longer baseline TAT and smaller sample size.

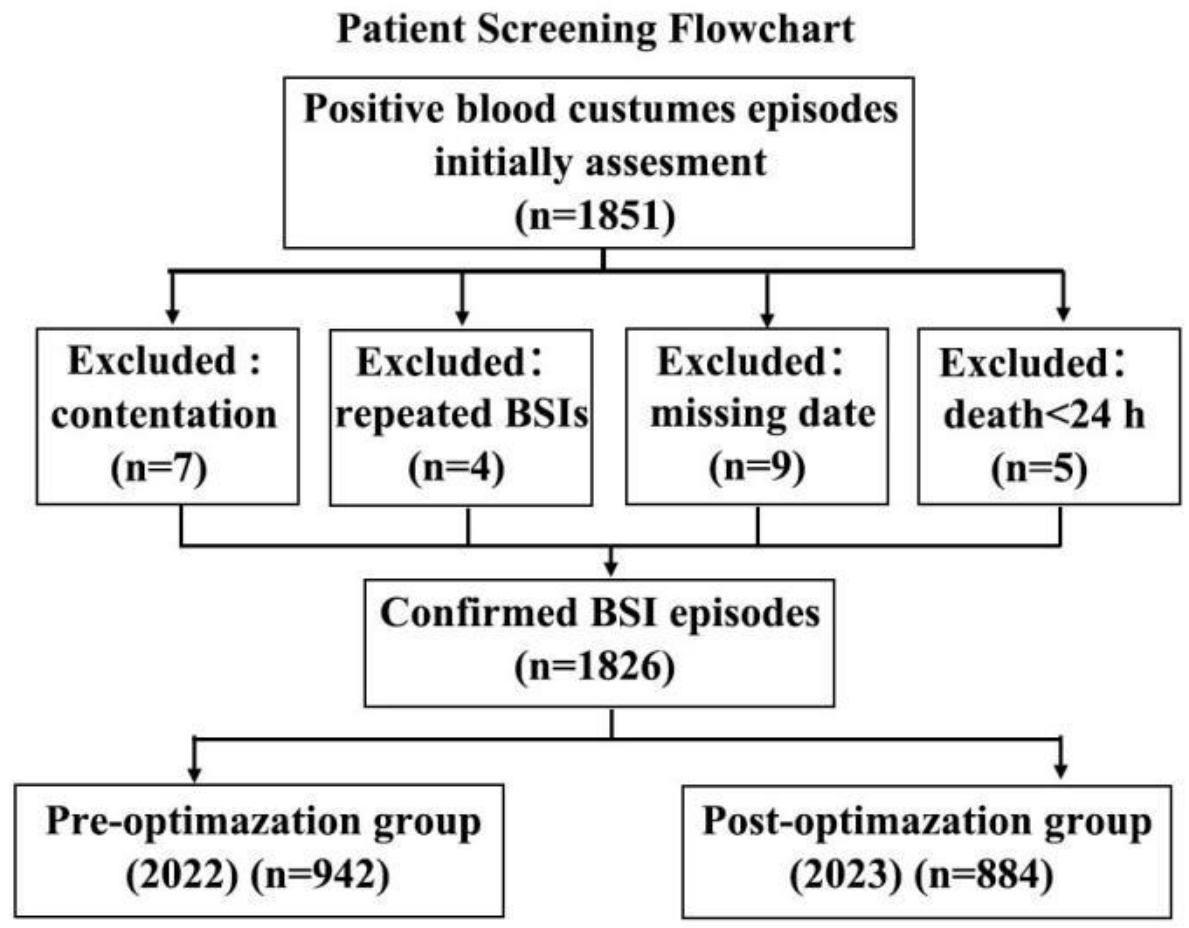

Supplementary Figure 1. The patient screening flowchart

Patient screening flowchart. Flow diagram illustrating the selection of bloodstream infection (BSI) episodes from all initially assessed positive blood cultures across 10 tertiary hospitals in Shandong Province. Cases were sequentially excluded for contamination, repeat episodes within the same hospitalization, incomplete core data, and death within 24 hours of admission. A total of 1,826 confirmed BSI episodes (942 pre-optimization, 884 post-optimization) were included in the final analysis.

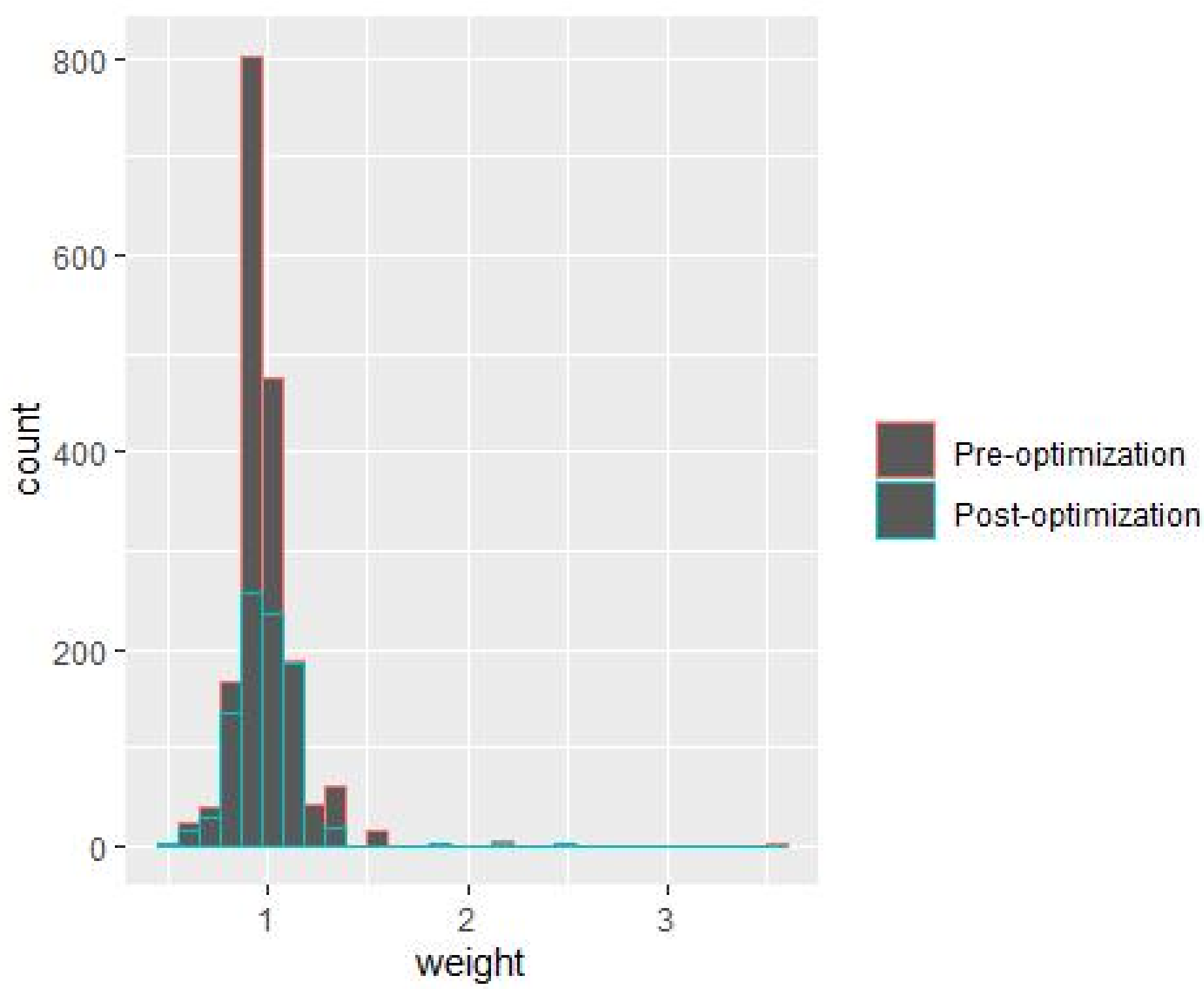

Supplementary Figure 2. Distribution of propensity score-based inverse probability weights

Shows the distribution of inverse probability weights for the pre-optimization and post-optimization groups. Good overlap between the two distributions with no extreme weights confirms that the positivity assumption is satisfied.
